# Engineered fluorescent proteins illuminate the bacterial periplasm

**DOI:** 10.5936/csbj.201210013

**Published:** 2012-11-22

**Authors:** Thorben Dammeyer, Philip Tinnefeld

**Affiliations:** a Institut für Physikalische und Theoretische Chemie, NanoBioSciences, Technische Universität Braunschweig, Hans Sommer Str. 10, 38106 Braunschweig, Germany

**Keywords:** bacterial export, fluorescent proteins, protein folding, chromophore maturation, sfGFP, periplasm

## Abstract

The bacterial periplasm is of special interest whenever cell factories are designed and engineered. Recombinantely produced proteins are targeted to the periplasmic space of Gram negative bacteria to take advantage of the authentic N-termini, disulfide bridge formation and easy accessibility for purification with less contaminating cellular proteins. The oxidizing environment of the periplasm promotes disulfide bridge formation - a prerequisite for proper folding of many proteins into their active conformation. In contrast, the most popular reporter protein in all of cell biology, Green Fluorescent Protein (GFP), remains inactive if translocated to the periplasmic space prior to folding. Here, the self-catalyzed chromophore maturation is blocked by formation of covalent oligomers via interchain disulfide bonds in the oxidizing environment. However, different protein engineering approaches addressing folding and stability of GFP resulted in improved proteins with enhanced folding properties. Recent studies describe GFP variants that are not only active if translocated in their folded form *via* the twin-arginine translocation (Tat) pathway, but actively fold in the periplasm following general secretory pathway (Sec) and signal recognition particle (SRP) mediated secretion. This mini-review highlights the progress that enables new insights into bacterial export and periplasmic protein organization, as well as new biotechnological applications combining the advantages of the periplasmic production and the *Aequorea*-based fluorescent reporter proteins.

## Introduction

Since its discovery in 1962 [1] and subsequent cloning of the wt-Green fluorescent protein in 1994 [2] the jellyfish *Aequorea* fluorescent proteins are the most widely used reporter proteins in all areas of biology. Due to their unique independence from cellular chaperones and non proteinogenic compounds (other than molecular oxygen) for chromophore maturation [3] they outcompete other genetically encoded but cofactor dependent fluorescent proteins like phycobiliproteins [4] and other dyes for many *in vivo* applications.

The long history of biotechnological exploration and engineering lead to a variety of available GFP variants with mutations affecting the spectral properties and the brightness by improving chromophore formation, folding or solubility as well as the discovery and engineering of related proteins from other organisms, e.g. the anthozoan mFruit-family proteins [5]. However, although features of many new fluorescent proteins meet or exceed some properties of enhanced GFP (eGFP), no single fluorescent protein has been discovered yet, that excels in all of them. The versatility of the GFP variants is a result of different optimization and selection strategies. Notable improvements were achieved by random mutagenesis and DNA shuffling on GFP or GFP circular permutants followed by screening for increased brightness of colonies or cells [6, 7]. With the increasing number of amino acid positions identified to influence specific properties, site directed mutagenesis approaches where applied more and more for fine-tuning of variants to meet the requirements for a chosen application. Site directed mutagenesis or consensus engineering [8] approaches are facilitated by the decline in gene synthesis prices, which easily allows the introduction of silent mutations for codon usage adaptation [6, 9, 10], restriction site elimination and other *in silico* modifications. Advanced engineering led to the availability of *Aequorea*-based fluorescent protein tools with surprising complexity like the multi colour bimolecular fluorescence complementation (BiFC)-system where split-FPs reconstitute to fluorescent proteins with altered spectroscopic properties [11–14] or the reversibly switchable fluorescent proteins rs-eGFP or dreiklang for super resolution microscopy, that can be reliably toggled on or off by illumination with different excitation wavelengths [15–17].

The structure of correctly folded GFP consists of an internal fluorophore surrounded by a tight beta-barrel [18]. Maturation of the intrinsic chromophore through cyclization and oxidation of the internal tri-peptide motif (Ser65-tyr66-Gly67) depends on the proper formation of the tight beta-barrel structure. Formation of the beta-barrel structure with an immature chromophore under anaerobic conditions and subsequent shift to higher oxygen concentration allows comparison of the oxygen dependent chromophore maturation kinetics of GFP variants independent of the folding process [19]. Despite the oxygen dependent chromophore maturation process [3], formation of active fluorescent protein is inhibited in oxidizing environments [20–23]. In the oxidizing endoplasmic reticulum (ER) of cells, approximately 50% of eGFP was found to be inactive [20, 24] and eGFP was found to be largely inactive in the bacterial periplasmic space [22]. GFP is a natively cytoplasmic protein and its formation is independent of intramolecular disulfide bonds. The two native cysteine residues C49, C71 are separated by 2.4 nm far out of range for disulfide bridging. During folding C49 and C71 are exposed and can potentially form interchain-disulfide bridges with other proteins or GFP folding intermediates [24], which are then trapped in a non-fluorescent state, where the chromophore maturation is hampered. The most utilized bacterial general secretory pathway (Sec) [25], which involves protein folding in the periplasm following secretion is thus not accessible for GFP and GFP fusion protein experiments, while the use of a second Tat (twin-arginine translocation) export pathway [26] capable of exporting proteins following folding in the cytoplasm yields periplasmic GFP fluorescence. However, given the importance of the bacterial periplasmic space for targeting of biotechnological and biomedical relevant proteins [27] that rely on the oxidizing environment for disulfide bridge formation and proper folding [28, 29], this is an important constraint for GFP applications. Furthermore, research on biological important processes, that happen largely in the “entrance hall” of the cell (e.g. like environmental signaling, cellular transport, antibiotic resistance mechanisms or cell devision), would benefit from a wide choice of sophisticated genetically encoded fluorescent markers with activity in the periplasm.

So far the folding properties of GFP variants have been exploited to report on the folding status of a fusion partner [30] or to stabilize and enhance solubility of a difficult fusion partner [31]. The maturation kinetics, influence studies of translation and translation-coupled localization, as well as other time dependent measurements [32, 33]. The folding and autocatalytic chromophore maturation characteristics of the GFP variants can therefore pose a problem for different specific applications and have been subject to extensive protein engineering and artificial evolution.

**Figure 1:**
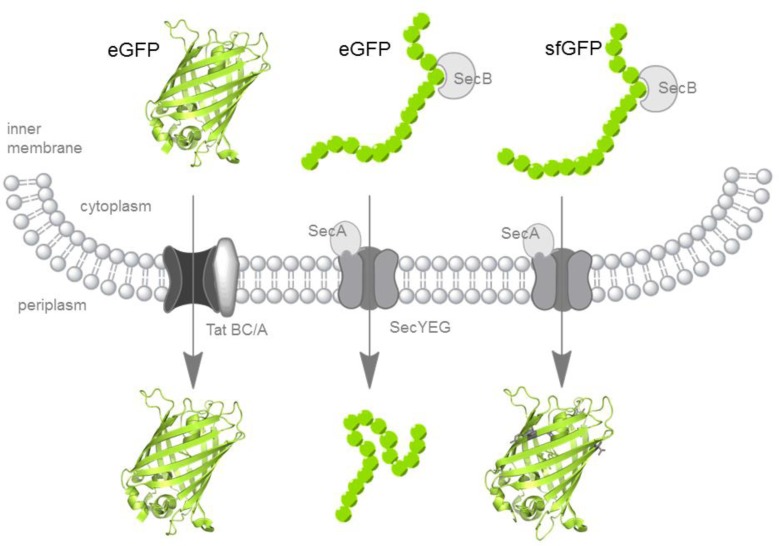
Schematic representation of bacterial export mechanisms and GFP-variants. Active eGFP (pdb 2Y0G [47]) folds in the cytoplasm and is exported in its folded state *via* the twin-arginine translocation (Tat-) pathway if targeted by Tat specific signal sequences (left). Targeted via the posttranslational Sec-pathway or the SRP dependent cotranslational branch (not depicted) eGFP is prevented from folding in the cytoplasm and translocated in its unfolded state. The oxidizing environment of the periplasm inhibits proper folding and chromophore maturation and GFP remains unfolded (mid). Unlike eGFP, sfGFP (pdb2B3P [34]) with the additional mutations S30R, Y39N, N105T, Y145F, I171V and A206V (residues in grey stick representation) yields active fluorescent protein in the periplasm following Sec mediated export (right).

Only very recently a super folder variant of GFP (sfGFP) [34] has been reported to be active in the bacterial periplasmic space even if translocated *via* posttranslational Sec or cotranslational SRP mediated transport [20, 21] – export pathways implying chromophore maturation in the periplasm (Figure 1, Table 1).

**Table 1:** Progress of GFP folding enhancement and its periplasmic export.

GFP variant	Finding	Translocon	Localization	Signal Sequence	Reference
fr(folding reporter)GFP^a^	Folding reporter assay for proteins fused to GFP	-	cytoplasm	-	[30]
GFPuv^b^	Reporter for protein localization in *E.coli*/ fluorescent in cytoplasm inactive in periplasm	SecYEG	cytoplasm	pre-MBP	[22]
GFPmut3* c	active GFP, translocated folded	Tat	periplasm	ssTorA	[40]
GFPmut2^d^	active GFP, translocated folded – (concentrates at the poles in reponse to osmotic up-shock)	Tat	periplasm	ssTorA	[41]
sf(super folder)GFP^e^	selected starting from frGFP screening for enhanced folding properties of insoluble ferritin frGFP fusions	-	cytoplasm	-	[34]
frGFP^a^; GFPmut2^d^	failed for Sec export and to fluoresce	SecYEG/SecYEG/SecYEG-SRP	cytoplasm	ssMBP/ssPhoA/ssDsbA	[23]
sfGFP^e^	sfGFP accumulated in cytoplasm	SecYEG/SecYEG/SecYEG-SRP	cytoplasm	ssMBP/ssPhoA/ssDsbA	[23]
ffGFP(P7)^f^	selected starting from GFPmut2 using Sec folding quality control	SecYEG-SRP	cytoplasm	ssDsbA	[23]
sfGFP^e^	sfGFP is functional *in vivo* at 70 degrees C	Tat	periplasm	PhoA	[42]
sfGFP^e^-fusions	fluorescent in bacterial periplasm	SecYEG-SRP	periplasm	ssDsbA	[43–45]
mGFP^g^	inactive in bacterial periplasm	SecYEG-SRP	periplasm	preMBP*1 [46]	[20]
sfGFP^e^	active in oxidizing environments	SecYEG-SRP	periplasm	preMBP*1 [46]	[20]
sfGFP^e^	active sfGFP is transported preferentially by the cotranslational SecYEG-SRP branch	SecYEG-SRP/SecYEG/SecYEG	periplasm/periplasm/cytoplasm	ssDsbA/pre-MBP /ssMBP	[21]

Amino acid mutations in the fluorescent protein sequence relative to wild type GFP for

a frGFP: F64L, S65T, F100S, M154T, V164A;

b GFPuv (cycle-3): F100S, M154T, V164A;

c GFPmut3

* S2R, S65G, S72A;

d GFPmut2 S65A, V68L, S72A;

e sfGFP: S65T, F64L, F100S, M154T, V164A, S30R, Y39N, N105T, Y145F, I171V, A206V;

f ffGFP(P7): F64L, S65A, V68L, S72A, N105Y, E124V, Y145F;

g mGFP (eGFP): S65T, F64L.

In this mini-review we summarize folding enhancement studies and their implications and applications with regard to bacterial export machineries.

## From folding reporter to superfolder GFP

The broad application range of GFP is the result of early engineering approaches and identification of beneficial mutations. Especially the S65T mutation improved the spectroscopic characteristics and fluorescence quantum yield [35], while the F64L mutation leads to eGFP with enhanced folding at 37°C. The cycle-3 mutants F100S, M154T, V164A further improved the fluorescence by reducing aggregation and increasing chromophore activation [6]. Based on eGFP with the cycle-3 mutations *Waldo et. al*. described a dependency of the fluorescence signal of protein fusions on the solubility and folding of proteins fused to the N-termini of GFPs [30]. From this discovery one of the most interesting early application of GFP in protein engineering and biotechnology, besides monitoring of expression [2, 32] arose. The observed dependency of chromophore maturation on the correct protein folding and thus ability of folding reporter GFP (frGFP) to report on the folding status of a protein from the living cell enabled directed evolution approaches by simple screening for increased fluorescence (Table 1). This has been exploited to screen target proteins with improved folding and solubility or to select soluble protein parts or domains suitable for crystallization. As the weak and insoluble expression of recombinant proteins and the formation of inclusion bodies is one of the major problems in protein sciences and biotechnology, this reporter assay was widely applied, adapted and iteratively improved [30, 36–39].

Several years later, a selection process similar as the one applied in the folding reporter studies to enhance folding and solubility of target proteins fused to GFP was utilized to improve folding of GFP itself (Table 1). Here, a ferritin-frGFP fusion underwent several cycles of DNA shuffling in order to select a fluorescent GFP variant that folds unaffected from the weakly folding fusion partner. The result was super folder GFP (sfGFP), which shows much faster maturation and folding kinetics due to the additional mutations S30R, Y39N, N105T, Y145F, I171V and A206V, while the spectroscopic properties and relative quantum efficiencies were only slightly changed compared to frGFP [34]. Like other largely soluble protein tags, this variant could even be used to increase solubility of proteins which are difficult to handle due to weak intrinsic solubility [31, 34].

## Presecretory quality control aids on selection of fast folding GFP variants

A completely different approach to select for GFP variants with improved folding characteristics was used by Fisher *et al* [23] (Table 1). Focusing on bacterial export and the quality control of secretion machineries, they hypothesized and tested a screening method for improved GFP folding characteristics based on the discriminative selection of the native SecYEG pathway quality control. Here, a slow or weakly folding protein is recognized and eliminated through degradation by a pathway intrinsic control mechanism, while a fast and stably folding variant is folded before secretion and resistant to unfolding resulting in an increased cellular fluorescence signal from active GFP in the cytoplasm. Controls targeted to the periplasm using the ssDsbA signal peptide could clearly distinguish between frGFP and sfGFP. A directed evolution approach and application of the new folding screening assay, accounting the cytoplasmic fluorescence accumulation for GFPs folding efficiency, led to the identification of fast folding GFP variants (e.g. the ffGFP P7).

## Green fluorescent protein targeted via the Tat pathway

Associated with bacterial export pathways, GFP was first introduced as a reporter protein suitable for protein localization studies in *E.coli*, based on the discovery that GFP is active in the cytoplasm and inactive if fused to pre-maltose binding protein for targeting *via* the Sec pathway (Table 1). Figure 1 schematically represents this failure of GFP to fold in the periplasm following Sec-pathway mediated export. Conversely to the established localization reporter proteins β-lactamase and alkaline phosphatase, proteins that are active only if exported to the bacterial periplasm, GFP behaved more similar to β-galactosidase which is only active in the cytoplasm [22].

The Tat (twin-arginine translocation) system is a bacterial protein export pathway distinct from the general secretory pathway (Sec) with the remarkable characteristic to mediate transport of largely or completely folded proteins across the cytoplasmic membrane. The substrates of this pathway are therefore predominantly proteins that require the incorporation of cofactors in the cytoplasm, need assistance of cytoplasmic chaperones for folding prior to export or are proteins that fold too fast and stable for the Sec intrinsic stabilization of the unfolded polypeptide state [26]. The Tat pathway substrates are targeted *via* signal sequences with a characteristic twin-arginine motif like the widely used ssTorA (Table 1) of the trimethylamine N-oxide (TMAO) reductase, a molybdenum cofactor containing protein.

Consequentially, two independent studies targeted GFP *via* ssTorA (Table 1) to the Tat-pathway to proof the hypothesis that readily folded proteins can be transported by this export mechanism. The successful detection of GFP fluorescence, located to the bacterial periplasmic space for the first time, was evidence for the new and substantially different export mechanism [40, 41]. GFP remained active in the bacterial periplasm if exported in a mature folded state, indicating that the previously observed periplasmic inactivity is indeed associated to an intrinsic folding and chromophore maturation error in the oxidizing periplasmic environment. Figure 1 schematically represents the successful Tat-mediated translocation of GFP.

## Sec-Pathway mediated export of super-folder GFP

The general secretory (Sec) pathway is the most utilized export pathway in almost all bacteria [25]. Presecretory substrate proteins pass through the SecYEG translocase and are exported in a posttranslational manner. The chaperone SecB binds posttranslationally or in late translation to the presecretory proteins and keeps the proteins in an unfolded or loosely folded conformation with the signal peptide accessible for interaction with the translocation machinery [48] as shown in Figure 1. A co-translational SecYEG mediated branch can be addressed via signal recognition particle (SRP) binding to the nascent polypeptide chain generally accomplished by more hydrophobic signal sequences [46]. For both pathways, proteins are threaded through the translocon in an unfolded state and subsequently fold in the periplasm [48]. Unlike the fluorescent mCherry which does not contain any cysteine residues GFPs and GFP fusion proteins are inactive if targeted to the bacterial periplasm *via* the Sec pathway. Notably, no disulfide bonded oligomers have been detected directely from the bacterial periplasm so far and detection of accumulated, non-fluorescent and Sec targeted eGFP in the cytoplasm [20] might also indicate an obstruction of the translocon. Nevertheless, the inactivity and the detection of misfolded oligomeric mixed disulfides of GFPs targeted to the secretory pathway of eukaryotic cells [20, 24] indicates that misfolding due to unspecific inter- or intra-molecular disulfide bond formation between folding intermediates occurs also upon bacterial secretion.

**Figure 2:**
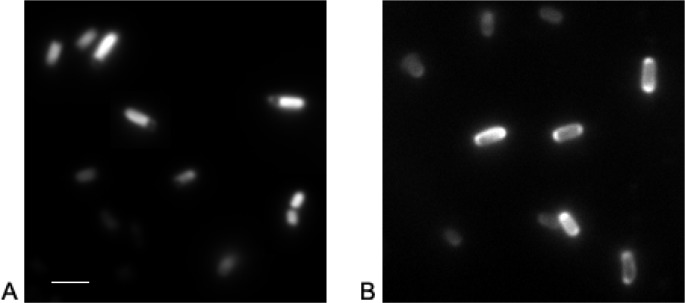
Gram negative bacteria producing a folding improved YFP. A) An engineered-YFP variant is fluorescent in the cytoplasm if expressed without a signal sequence and B) actively folds following Sec-mediated export to the periplasm as evidenced by the halo like peripheral fluorescence signal in *Escherichia coli* BL21 cells (unpublished results of the authors, scale bar, 2.5 µm).

Super folder GFP (sfGFP), however, is able to fold and mature into its fluorescent conformation unaffected from the periplasmic environment [20, 21], as depicted in the scheme of Figure 1 (Table 1). We could demonstrate for the first time that this holds also true for an engineered monomeric yellow color variant as shown in Figure 2. Two of the super folder mutations (S30R and Y39N) are found ahead of the cysteine residues in the primary sequence and consequently will be secreted earlier than either of the cysteines. Both mutations have been shown to alter the conformation of the first three β-strands providing the most significant improvements of sfGFPs folding robustness [34]. The folding of the first three β-strands therefore is likely to be critical for GFP folding while the peptide chain emerges from the translocation channel [20]. Only the improved folding of the β-barrel enables the intact autocatalytic chromophore formation in the bacterial periplasmic space.

## Outlook

The discovery of the super folder GFP mutations and the folding robustness they confer to GFP allows maturation in the periplasm and enabled diverse new applications. Periplasmic protein localization studies using GFP, previously only possible by targeting the analyzed proteins *via* the Tat pathway, can now be performed recruiting the native signal sequences and exportable sfGFP as demonstrated e.g. for EnvC and Pal [21]. Furthermore, periplasmic colocalization studies e.g. with mCherry and sfGFP fusion proteins are now feasible [49] making the periplasm amenable for new labeling approaches and consequently advanced imaging applications. Moreover, the design of biosensors using receptor fluorescent protein fusions is simplified. SfGFP have been shown to enhance fusion protein solubility [34] a property which in combination with the unbiased folding in the periplasm represents a valuable improvement for recombinant biotechnological protein production. Especially the many biotechnological and biomedical relevant proteins like the repertoire of recombinant antibody fragments that require disulfide bond formation [28, 29, 50] can now be produced as GFP fusions targeted to the periplasm. Those fusions proteins can subsequently easily be extracted as ready labeled active probes for multi-purpose use. Moreover, the work with superfolder GFP is clear evidence that there is significantly more room for engineering improvements even in the highly optimized GFP derivatives and continued efforts using directed evolution approaches will without doubts lead to further optimized variants with improved characteristics like spectral properties, photostability, brightness, acid resistance and utility as tags for cellular imaging.
